# Long-Term Biochar Application Enhances Peanut Yield by Delaying Leaf Senescence and Optimizing Nutrient Balance

**DOI:** 10.3390/plants15132025

**Published:** 2026-06-30

**Authors:** Xinyu Mu, Ni Zhang, Wentao Yu, Meng Zhang, Ning Liu, Na Zhang, Shunguo Liu, Xiumei Zhan

**Affiliations:** 1 College of Land and Environment, Shenyang Agricultural University, Shenyang 110866, China; 2024240619@stu.syau.edu.cn (X.M.); zn903164341@163.com (N.Z.); tiao0912@163.com (W.Y.); 15940707511@163.com (M.Z.); lnbrisk@163.com (N.L.); 2 Liaoning Agricultural and Rural Development Service Center, Shenyang 110034, China; gbzxflk@163.com (N.Z.); lnsspb@163.com (S.L.)

**Keywords:** biochar, peanut, leaf senescence, nutrient balance, nutrient ratio

## Abstract

Premature leaf senescence during the late growth stage of *Arachis hypogaea* (peanut) reduces photosynthetic capacity and restricts pod filling, thereby limiting yield. Although biochar has been widely used for soil improvement due to its porous structure and stability, its long-term effects on sustaining photosynthetic performance and improving yield through nutrient regulation remain insufficiently understood. Based on a long-term field micro-plot experiment established in 2009, four fertilization treatments were evaluated: maize straw return + NPK (CS), pig manure compost + NPK (PMC), biochar + NPK (BIO), and biochar-based fertilizer (BF), with three replicates. Peanut was used as the test crop in 2024. Photosynthetic parameters, SPAD values, leaf *N*, *P*, and *K* contents, nutrient use efficiencies (PNUE, PPUE, PKUE), nutrient ratios, carbon allocation characteristics, and yield were measured at key growth stages. Results showed that the BIO treatment maintained higher net photosynthetic rate and SPAD value at the full-pod maturity stage compared with CS, PMC, and BF. Leaf *K* content was significantly increased under BIO, while the *N*:*K* ratio remained below 2.1, indicating reduced risk of potassium-related nutrient imbalance. The *N*:*P* ratio also remained relatively stable, suggesting improved nutrient balance with respect to phosphorus during the late growth stage. BIO showed the highest estimated contribution of photosynthetic carbon to pod dry weight, and yield increased by 19.2–28.6% compared with other treatments. These results indicate that long-term biochar application was associated with sustained late-stage photosynthetic performance and improved leaf nutrient balance, which may contribute to higher peanut yield. This study provides new evidence supporting the potential role of long-term biochar application in sustainable peanut production.

## 1. Introduction

*Arachis hypogaea* (peanut) is an important oilseed and economic crop in China and plays a vital role in agricultural production. Leaves are the primary organs responsible for photosynthesis in peanut, and the duration of their functional period directly determines dry matter accumulation and pod yield formation. However, premature leaf senescence commonly occurs during the late growth stage of peanut production, leading to a decline in photosynthetic function and insufficient seed filling, which severely restricts the realization of yield potential and ultimately affects yield formation [1]. Therefore, delaying leaf senescence and maintaining photosynthetic capacity during the late growth stage through rational fertilization management are of great importance for achieving high-yield peanut cultivation [2].

Biochar is characterized by a rich porous structure, large specific surface area, strong adsorption capacity, and high chemical stability, and has therefore been widely applied in soil improvement, carbon sequestration, and emission reduction [3]. At the level of crop photosynthetic physiology, biochar application can also markedly affect photosynthesis. Previous studies have demonstrated that biochar can enhance photosynthetic rate and leaf chlorophyll content by mitigating the interference of salinity with physiological processes [4]. However, most existing studies have focused on short-term effects, and systematic evidence remains limited regarding whether long-term field application of biochar can continuously delay peanut leaf senescence and increase yield.

More importantly, different organic materials, including straw, pig manure, biochar, and biochar-based fertilizer, differ substantially in chemical composition and decomposition characteristics; therefore, their long-term application may affect crop photosynthetic function and senescence processes through distinct mechanisms. Conventional organic materials, such as straw and pig manure, are readily decomposable and can release nutrients rapidly, but their nutrient supply is often less persistent [5]. In contrast, biochar is highly aromatic and chemically stable, allowing it to persist in soil over the long term [6]. Straw return can promote early vegetative growth by rapidly supplying nutrients [7], but insufficient nutrient availability at later growth stages may induce premature senescence. Pig manure provides a broad spectrum of nutrients, but its nutrient release rate is regulated by microbial activity [8]. Biochar, however, may ensure a more stable nutrient supply throughout the entire growth period through slow nutrient release and nutrient adsorption–desorption equilibrium. As a compound product of biochar and chemical fertilizer, biochar-based fertilizer combines both rapid-acting and slow-release properties [9]. Nevertheless, few studies have systematically compared the effects of different organic materials on peanut leaf senescence and photosynthetic function within the same long-term field experimental platform.

High crop yield depends not only on the sufficient supply of individual nutrients, but also on the relative balance of nutrient availability; in other words, crop yield is closely associated with both the quantitative adequacy and proportional balance of nutrient supply. Ratios such as *N*:*P* and *N*:*K* in plant tissues are key indicators for identifying nutrient limitations to plant growth and metabolic strategies [10]. Previous studies have shown that an increase in the leaf *N*:*P* ratio in forest plants induces phosphorus limitation, thereby reducing photosynthetic rate and photosynthetic phosphorus-use efficiency (PPUE) [11]. In agroecosystems, the leaf *N*:*K* ratio in maize is significantly negatively correlated with net photosynthetic rate, indicating that potassium deficiency restricts the transport of photosynthetic assimilates [12]. Therefore, crop photosynthetic function is regulated not only by the content of a single nutrient, but also by the overall nutrient balance. In peanut production, phosphorus and potassium supply often becomes insufficient during the late growth stage. Although previous studies have examined changes in crop nutrient concentrations following biochar addition [13,14,15], few have systematically investigated whether long-term biochar application can alleviate nutrient limitation by optimizing leaf nutrient stoichiometry, thereby regulating photosynthetic function and the senescence process. In particular, the role of potassium in delaying peanut senescence has often been overlooked, and whether biochar can alleviate potassium limitation as a key mechanism for yield improvement warrants further investigation.

This study was based on a long-term field experiment initiated in 2009 at Shenyang Agricultural University, including four fertilization treatments: biochar combined with NPK fertilizer (BIO), biochar-based fertilizer (BF), crop straw return combined with NPK fertilizer (CS), and pig manure compost combined with NPK fertilizer (PMC). Among these treatments, BIO represented direct biochar amendment, whereas BF represented a fertilizer product containing biochar. The CS treatment represented the conventional maize straw return practice commonly adopted in the study region and was used as the reference treatment for comparison. Unlike previous studies that mainly focused on crop yield responses or soil nutrient improvement following biochar application, this study integrated late-stage photosynthetic maintenance, leaf *N*,*P*,*K* nutrient balances, nutrient limitation diagnosis, carbon allocation, and yield formation within a long-term field experimental platform, providing a more comprehensive understanding of the physiological and ecological processes associated with yield improvement. Photosynthetic parameters, including net photosynthetic rate (*Pn*), stomatal conductance (*Gs*), intercellular CO_2_ concentration (*Ci*), and stomatal limitation value (*Ls*), SPAD values (an indirect and non-destructive indicator of leaf greenness and chlorophyll status), leaf nitrogen, phosphorus, and potassium contents, and photosynthetic nutrient-use efficiency were systematically measured at four peanut growth stages, including the seedling stage, flowering and pegging stage, pod-setting stage, and pod-filling to maturity stage. Leaf nutrient ratios (*N*:*P*, *N*:*K*, and *K*:*P*) were also calculated, and carbon allocation characteristics were integrated to explore potential relationships among photosynthetic performance, nutrient balance, and yield formation under long-term biochar application. Specifically, this study aimed to address the following scientific questions: (1) Does long-term biochar application delay peanut leaf senescence and maintain photosynthetic capacity during the late growth stage more effectively than other organic materials? (2) Does biochar alleviate potassium and phosphorus limitations during the late growth stage by optimizing leaf nutrient ratios? (3) Do the above pathways involving delayed senescence, alleviated nutrient limitation, and sustained photosynthesis ultimately drive significant yield improvement? The findings of this study may provide a theoretical basis for green and efficient peanut cultivation and the resource utilization of biochar.

## 2. Materials and Methods

### 2.1. Experimental Description

The field micro-plot experiment was conducted at the National Peanut Industry Technology System Soil and Fertilizer Long-term Experimental Station of Shenyang Agricultural University (40°48′ N, 123°33′ E). The site has a temperate monsoon climate, characterized by dry springs and humid conditions from June to August. The annual mean temperature ranges from 7.0 to 8.1 °C, and the annual mean precipitation ranges from 574 to 684 mm. The soil type is sandy clay loam. The basic physicochemical properties of the 0–20 cm soil layer before the experiment were as follows: The soil organic matter content was 13.3 g kg^−1^. The total nitrogen content was 0.86 g kg^−1^. The alkaline hydrolyzable nitrogen content was 51.3 mg kg^−1^. The total phosphorus content was 0.51 g kg^−1^. The available phosphorus content was 4.6 mg kg^−1^. The available potassium content was 114.5 mg kg^−1^. The soil pH was 6.1. Soil organic matter was determined using the potassium dichromate oxidation method. Total nitrogen was determined using the Kjeldahl method. Alkaline hydrolyzable nitrogen was determined using the alkaline hydrolysis diffusion method. Total phosphorus was determined after acid digestion using the molybdenum blue colorimetric method. Soil available phosphorus was extracted with 0.5 mol L^−1^ NaHCO_3_ (Olsen method) and determined colorimetrically. Soil available potassium was extracted with 1 mol L^−1^ NH_4_OAc and measured by flame photometry. Soil pH was determined potentiometrically in a soil-to-water suspension at a ratio of 1:2.5 (*w*/*v*) [16].

### 2.2. Experimental Design

The long-term experiment began in 2009 and included four treatments. The experiment was arranged as a randomized complete block design with three independent field microplots for each treatment. Each microplot was regarded as an experimental unit in the statistical analyses. Treatment 1: Maize straw returning at 4500 kg ha^−1^ + NPK (CS); Treatment 2: Pig manure compost at 4000 kg ha^−1^ + NPK (PMC); Treatment 3: Biochar at 1500 kg ha^−1^ + NPK (BIO); Treatment 4: Biochar-based fertilizer (10-13-13) at 750 kg ha^−1^ (BF). The application rates of N, P, and K were 75, 42.6, and 80.9 kg ha^−1^, respectively. The total amounts of N, P, and K were kept consistent across the four treatments; that is, the amount of supplemental chemical fertilizer was calculated as the total nutrient requirement minus the nutrients contributed by the organic materials applied each year. The biochar particles were produced from corncob pyrolyzed at 450 °C, then sieved through 80–100 mesh and pelletized. The biochar-based fertilizer consisted of inorganic fertilizers compounded with biochar produced from maize straw through pyrolysis at 450–550 °C. Compared with the BIO treatment, in which biochar and mineral fertilizers were applied separately, BF represented a co-granulated product integrating biochar and nutrients into a single fertilizer granule. Consequently, the two treatments differed not only in biochar application mode but also potentially in nutrient-release dynamics, nutrient availability, and carbon input characteristics throughout the growing season. The straw was crushed to 2–3 cm length. Both straw and pig manure compost were applied on a dry-weight basis. Each year, before sowing, all organic materials and fertilizers were uniformly incorporated into the 0–20 cm soil layer. The experiment was arranged in a randomized complete block design with three replicates. Each microplot measured 2 m^2^ and was separated by 20-cm cement barriers to prevent lateral movement of water and nutrients. *Arachis hypogaea* (peanut) were planted in a wide-ridge double-row pattern, with a plant spacing of approximately 11 cm and three plants per hill. The peanut variety used was *Arachis hypogaea* L. cv. Fuhua 12 150GY, a cultivar developed in China. Peanut was sown on 11 May 2024 and harvested on 1 October 2024. Table 1 presents the nutrient contents of the added organic materials.

### 2.3. Measurement Items and Methods

For each treatment, photosynthetic characteristics of functional leaves (the third leaf from the top of the main stem) were measured on clear, cloudless days between 9:30 a.m. and 12:00 p.m. at the seedling stage (35 DAS), flowering–pegging stage (59 DAS), podding stage (84 DAS), and pod-filling and maturity stage (117 DAS) in 2024. A Li-6400 portable photosynthesis system (LI-COR Inc., Lincoln, NE, USA) equipped with a standard 6 cm^2^ leaf chamber was used with an open gas path under natural light conditions. To minimize environmental variation, all treatments were measured within the same time period on each sampling date. The leaf chamber temperature was maintained at 25 °C, and the ambient CO_2_ concentration was 385 ± 5 μmol mol^−1^. Light-response curves were not determined in the present study; therefore, potential variation associated with natural irradiance should be considered when interpreting the gas-exchange data. Healthy and uniformly growing functional leaves were selected for measurement. Three representative plants were selected from each plot, and one functional leaf (the third leaf from the top of the main stem) was measured per plant. SPAD values were measured using a SPAD-502 m (Konica Minolta, Tokyo, Japan). Three readings were taken from the central portion of each selected leaf and averaged to obtain the SPAD value. SPAD was used as an indirect indicator of leaf greenness and relative chlorophyll status rather than a direct measurement of chlorophyll concentration. Plant samples were collected at each of the four growth stages (seedling, flowering and pegging, pod-setting, and pod-filling to maturity). At each sampling stage, three plants were randomly selected from each microplot, with sampling conducted from the central area to minimize border effects. Samples were separated into different plant organs when necessary, oven-killed at 105 °C for 30 min, then dried at 60 °C to constant weight, ground into powder, and stored for subsequent analysis. Biomass data obtained at the pod-setting and pod-filling to maturity stages were further used for estimating carbon translocation amount (CTA) and estimated photosynthetic carbon contribution (EPC). At harvest, peanut pod yield, 100-pod weight, and 100-kernel weight were determined from the entire micro-plot.

Calculation methods: After the pods were air-dried to a moisture content below 10%, 100 healthy pods with complete morphology and no damage, disease, or insect injury were randomly selected from the subsampled material and weighed. This value was recorded as the 100-pod weight. The selected 100 pods were then manually shelled. After shelling, 100 full and intact kernels were randomly selected and weighed. This value was recorded as the 100-kernel weight. The kernel rate was calculated as follows:

(1) $$\begin{array}{c} \mathrm{Kernel}\;\mathrm{percentage}\;\left(\%\right)=total\;kernel\;weight/total\;pod\;weight\times 100\; \end{array}$$

Plant nitrogen, phosphorus, and potassium were measured using the following methods: plant samples were digested with H_2_SO_4_–H_2_O_2_. Total nitrogen was determined using a Kjeldahl nitrogen analyzer. Total phosphorus was determined using the vanadium–molybdenum yellow colorimetric method. Total potassium was determined using flame photometry [17]. Photosynthetic nitrogen (phosphorus, potassium) use efficiency (PNUE, PPUE, PKUE; μmol·g^−1^·s^−1^) was calculated as follows:

(2) $$\mathrm{PNUE}\;\left(\mathrm{PPUE},\mathrm{PKUE}\right)=\frac{\mathrm{Net}\;\mathrm{photosynthetic}\;\mathrm{rate}\;\left(Pn\right)}{\mathrm{Nutrient}\;\mathrm{content}\;\left(N,P,K\right)}$$

Leaf nutrient ratios (*N*:*P*, *N*:*K*, and *K*:*P*) were calculated based on leaf nutrient contents and used to evaluate nutrient balance and plant nutritional status during different growth stages of peanut. The threshold values adopted in this study were derived from previous studies on plant nutrient ratios and empirical observations across different plant species and ecosystems. Because these thresholds were not specifically developed for peanut under fertilized field conditions, they were used only as comparative indicators of nutrient balance and relative nutrient demand among treatments and growth stages, rather than as definitive diagnostic criteria for nutrient limitation. Therefore, interpretations based on *N*:*P*, *N*:*K*, and *K*:*P* ratios should be regarded as relative assessments of nutritional status and nutrient imbalance rather than direct evidence of nutrient limitation.

### 2.4. Data Processing and Calculation Methods

Data were organized using Microsoft Excel 2010. Prior to statistical analysis, data normality and homogeneity of variance were evaluated using the Shapiro–Wilk test and Levene’s test, respectively. Because the primary objective of this study was to compare treatment effects within individual growth stages, statistical analyses were conducted separately for each sampling stage using one-way analysis of variance (ANOVA) followed by Duncan’s multiple range test in SPSS 19.0. Statistical significance was set at *p* < 0.05. Growth stages were analyzed independently because each sampling stage represented a distinct physiological phase of peanut development, and treatment effects at individual stages were the primary focus of this study.

For key treatment comparisons (BIO vs. CS, PMC, and BF), standardized effect sizes (Hedges’ g) and corresponding 95% confidence intervals (95% CI) were calculated to evaluate the magnitude and direction of treatment effects. Hedges’ g was calculated as follows:

(3) $$g=J\times \frac{\left(M_{1}-M_{2}\right)}{S_{P}}$$

where M_1_ and M_2_ are treatment means, S_p_ is the pooled standard deviation, and J is the small-sample correction factor. According to previous studies, effect sizes of 0.2, 0.5, and 0.8 were considered small, medium, and large effect sizes, respectively. Because the number of replicates was limited (*n* = 3), effect sizes and confidence intervals were interpreted cautiously and used primarily to describe the magnitude and direction of treatment differences rather than to provide definitive statistical inference [18].

Redundancy analysis (RDA) was conducted using Canoco 5.0 based on 12 field observations (four treatments × three replicates). Yield, carbon translocation, and estimated photosynthetic carbon contribution were used as response variables, whereas nine explanatory variables (*Pn*, *Gs*, *Ci*, *Tr*, SPAD, PNUE, PPUE, *N*:*P*, and *N*:*K*) were included as explanatory variables. Forward selection was used to evaluate the contribution of explanatory variables. The significance of the ordination model was assessed using Monte Carlo permutation tests with 499 unrestricted permutations [19].

Stomatal limitation value:

(4) $$Ls=1-\frac{C_{i}}{C_{a}}$$

Dry matter-based carbon transport estimate:

(5) $$C_{transport}=W_{\mathrm{stem}+\mathrm{leaf},\mathrm{pod}\text{-}\mathrm{setting}}-W_{\mathrm{stem}+\mathrm{leaf},\mathrm{pod}\text{-}\mathrm{filling}\;\mathrm{to}\;\mathrm{maturity}}$$

Dry matter-based photosynthetic carbon estimate:

(6) $$C_{photo}=W_{\mathrm{pod},\mathrm{mature}}-C_{\mathrm{transport}}$$

Because direct carbon flux measurements were not conducted in the present study, carbon transport amount and estimated photosynthetic carbon contribution were estimated using changes in organ dry matter between the pod-setting and maturity stages. These parameters represent relative estimates of carbon allocation patterns among treatments and should not be interpreted as direct measurements of newly assimilated carbon transport or allocation.

## 3. Results and Analysis

### 3.1. Dynamic Effects of Different Fertilization Treatments on Photosynthetic Parameters in Peanut

#### 3.1.1. Temporal Changes and Treatment Differences in Net Photosynthetic Rate (Pn)

**Figure 1:**
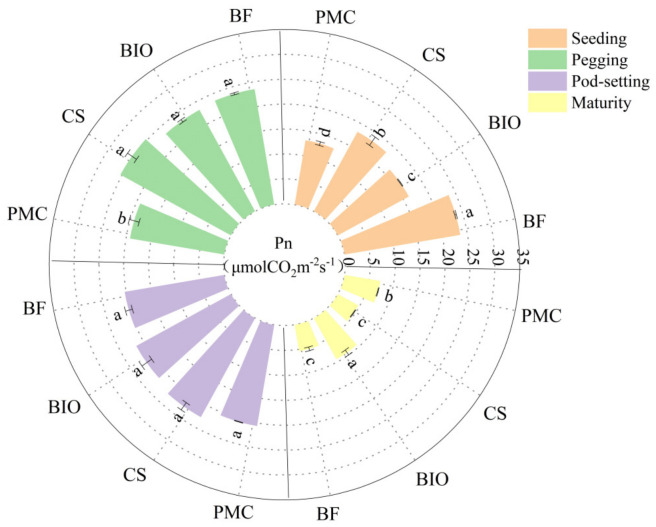
Net photosynthetic rate (*Pn*) of *Arachis hypogaea* (peanut) leaves under four fertilization treatments at four growth stages. Data were collected from a long-term field micro-plot experiment established at Shenyang Agricultural University, China. Measurements were conducted at the seedling stage, flowering and pegging stage, pod-setting stage, and pod-filling to maturity stage. BF, biochar-based fertilizer; BIO, biochar + NPK fertilizer; CS, corn straw return + NPK fertilizer; PMC, pig manure compost + NPK fertilizer. Different lowercase letters indicate significant differences among treatments at the same growth stage (*p* < 0.05).

As shown in Figure 1, the photosynthetic rate of peanut leaves varied markedly across growth stages, exhibiting an initial increase followed by a subsequent decline, with the advantage of BIO being most pronounced at the pod-filling to maturity stage. At the seedling stage, the photosynthetic rate under BF was significantly higher than that under the other treatments (*p* < 0.05), reaching 23.5 μmol CO_2_ m^−2^ s^−1^; however, it decreased by 62% by the pod-filling to maturity stage, reaching only 8.82 μmol CO_2_ m^−2^ s^−1^. In contrast, *Pn* under BIO at the same stage was 12.85 μmol CO_2_ m^−2^ s^−1^, which was significantly higher than that under CS (8.12), PMC (10.7), and BF (8.82) (*p* < 0.05). These results indicate that long-term biochar application effectively slowed the decline in photosynthetic rate during the late growth stage of peanut and delayed the deterioration of leaf function, whereas leaf senescence progressed more rapidly under the biochar-based fertilizer and straw treatments during the late growth stage.

#### 3.1.2. Changes in Stomatal Conductance (Gs) and Transpiration Rate (Tr)

**Figure 2:**
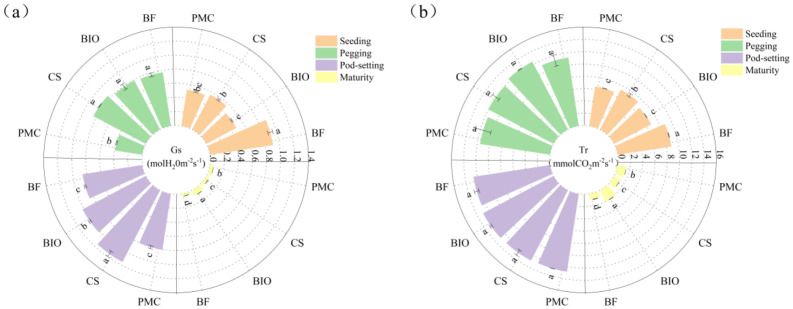
Stomatal conductance (*Gs*) and transpiration rate (*Tr*) of peanut leaves under four fertilization treatments at four growth stages. Data were collected from a long-term field micro-plot experiment established at Shenyang Agricultural University, China. Measurements were conducted at the seedling stage, flowering and pegging stage, pod-setting stage, and pod-filling to maturity stage. (**a**) stomatal conductance (*Gs*); (**b**) transpiration rate (*Tr*). *Gs*, stomatal conductance; *Tr*, transpiration rate. BF, biochar-based fertilizer; BIO, biochar + NPK fertilizer; CS, corn straw return + NPK fertilizer; PMC, pig manure compost + NPK fertilizer. Different lowercase letters indicate significant differences among different fertilization treatments at the same growth stage, as determined by analysis of variance (ANOVA) followed by Duncan’s test (*p* < 0.05).

Stomatal conductance reflects the opening and closure of leaf stomata and affects photosynthesis, respiration, and transpiration. As shown in Figure 2a, stomatal opening was greatest at the pod-setting stage among the different growth stages. In particular, *Gs* under CS reached 1.15 mol H_2_O m^−2^ s^−1^, which was significantly higher than that under the other treatments (*p* < 0.05). However, it sharply decreased to 0.13 mol H_2_O m^−2^ s^−1^ at the pod-filling to maturity stage. At the same stage, *Gs* under BIO was 0.21 mol H_2_O m^−2^ s^−1^; although lower than that at the pod-setting stage, it remained at a relatively high level, and *Tr* was also higher than that under the other treatments. Across growth stages, the variation in transpiration rate of peanut leaves under different fertilization treatments was generally consistent with that of photosynthetic rate, indicating that higher photosynthetic rates were accompanied by stronger transpiration rates. These results suggest that BIO maintained a relatively favorable degree of stomatal opening during the late growth stage, thereby facilitating CO_2_ exchange and sustaining photosynthetic activity.

#### 3.1.3. Relationship Between Intercellular CO_2_ Concentration (Ci) and Stomatal Limitation Value (Ls)

**Figure 3:**
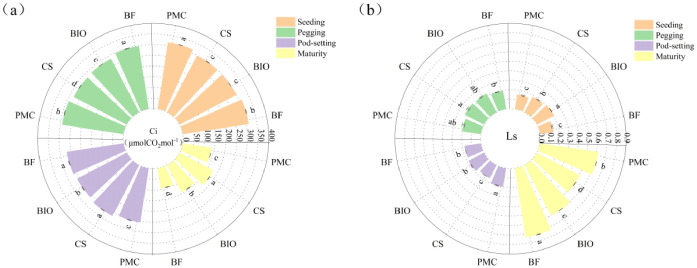
Intercellular CO_2_ concentration (*Ci*) and stomatal limitation value (*Ls*) of peanut leaves under four fertilization treatments. Data were collected from a long-term field micro-plot experiment established at Shenyang Agricultural University, China. Measurements were conducted at the seedling stage, flowering and pegging stage, pod-setting stage, and pod-filling to maturity stage. (**a**) intercellular CO_2_ concentration (*Ci*); (**b**) stomatal limitation value (*Ls*). *Ci*, intercellular CO_2_ concentration; *Ls*, stomatal limitation value. BF, biochar-based fertilizer; BIO, biochar + NPK fertilizer; CS, corn straw return + NPK fertilizer; PMC, pig manure compost + NPK fertilizer. Different lowercase letters indicate significant differences among different fertilization treatments at the same growth stage, as determined by analysis of variance (ANOVA) followed by Duncan’s test (*p* < 0.05).

Intercellular CO_2_ concentration (*Ci*) reflects the capacity of plant cells to assimilate CO_2_. As shown in Figure 3a, *Ci* generally decreased across the different growth stages. Fertilization treatments had certain effects on *Ci*, with significant differences observed at the pod-filling to maturity stage (*p* < 0.05). At the flowering and pegging stage, BF exhibited the highest *Ci*, reaching 297.9 μmol CO_2_ m^−2^ s^−1^, which was significantly higher than that under the other treatments. PMC had the second-highest *Ci*, at 290.4 μmol CO_2_ m^−2^ s^−1^, whereas BIO showed a relatively low *Ci* of only 228 μmol CO_2_ m^−2^ s^−1^.

Stomatal limitation (*Ls*) is regulated by changes in stomatal conductance and occurs when the amount of CO_2_ entering through the stomata becomes insufficient to meet the requirements for photosynthesis. As shown in Figure 3b, *Ls* differed significantly among treatments (*p* < 0.05), and all treatments exhibited a substantial increase in *Ls* at the pod-filling to maturity stage. Previous studies have shown that when *Pn* and *Ci* change in opposite directions, with *Ci* increasing and *Ls* decreasing, the reduction in *Pn* is mainly attributable to non-stomatal factors. Conversely, when *Pn* and *Ci* change in the same direction, with *Ci* decreasing and *Ls* increasing, the decline in *Pn* is mainly caused by stomatal factors associated with stomatal closure. These results indicate that the decrease in photosynthetic rate in peanut leaves at the pod-filling to maturity stage was primarily driven by stomatal factors. At this stage, CS showed both a low *Ls* and a low photosynthetic rate, whereas *Ci* was relatively increased, suggesting that the decline in *Pn* under CS was mainly caused by non-stomatal factors. BF exhibited an extremely high *Ls* of 0.74 but a very low *Pn*, indicating severe stomatal limitation. In contrast, BIO maintained a relatively low *Ci* and a high *Ls* of 0.58, suggesting that late-stage leaf senescence under BIO was mainly associated with a more coordinated relationship between stomatal limitation and intercellular CO_2_ concentration, reflected by a relatively balanced *Ls* and *Ci* pattern, which may help maintain CO_2_ supply for photosynthesis.

### 3.2. Effects of Different Fertilization Treatments on Leaf SPAD Value

**Figure 4:**
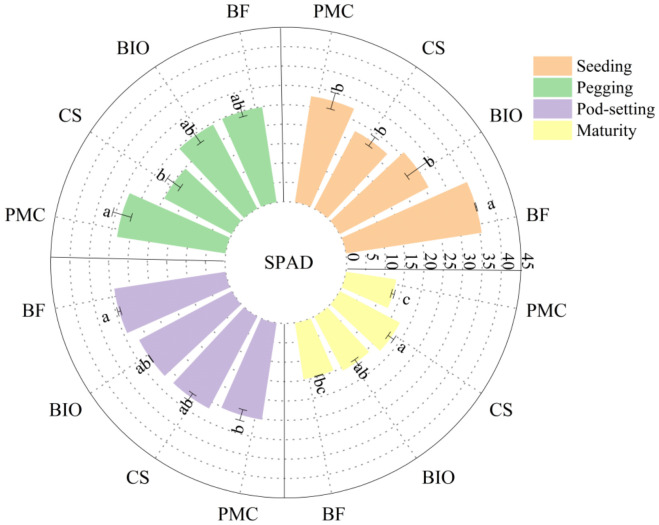
SPAD value of peanut leaves under four fertilization treatments. Data were collected from a long-term field micro-plot experiment established at Shenyang Agricultural University, China. Measurements were conducted at the seedling stage, flowering and pegging stage, pod-setting stage, and pod-filling to maturity stage. BF, biochar-based fertilizer; BIO, biochar + NPK fertilizer; CS, corn straw return + NPK fertilizer; PMC, pig manure compost + NPK fertilizer. Different lowercase letters indicate significant differences among different fertilization treatments at the same growth stage, as determined by analysis of variance (ANOVA) followed by Duncan’s test (*p* < 0.05).

As shown in Figure 4, the SPAD value of peanut leaves generally decreased with growth stage, and significant differences were observed among treatments at different growth stages (*p* < 0.05). At the seedling stage, BF showed a relatively high SPAD value of 35.5, but exhibited the greatest decline at the later stage, with a reduction of 58.6%. The SPAD value under BIO was relatively low at the early growth stage, at 25.0, but remained more stable during the later growth stages, with a slower decline of only 38.9%, indicating a clear advantage in delaying leaf senescence. PMC showed a relatively high SPAD value at the early growth stage, at 27.7, but decreased markedly at the later stage to only 13.1, representing a decline of 52.7%. CS had a relatively low SPAD value at the early growth stage, reaching only 22.6, but remained relatively stable throughout the growth period, with a smaller decline. Overall, BIO showed a relatively stable SPAD value across the growth period, with a smaller decline compared with BF and PMC. This pattern could indicate a tendency to maintain the chlorophyll content of the leaves even in later growth stages.

### 3.3. Effects of Different Fertilization Treatments on Leaf Nutrient Contents

#### 3.3.1. Temporal Changes and Treatment Differences in Leaf Nutrient Contents and Photosynthetic Nutrient Use Efficiencies

**Figure 5:**
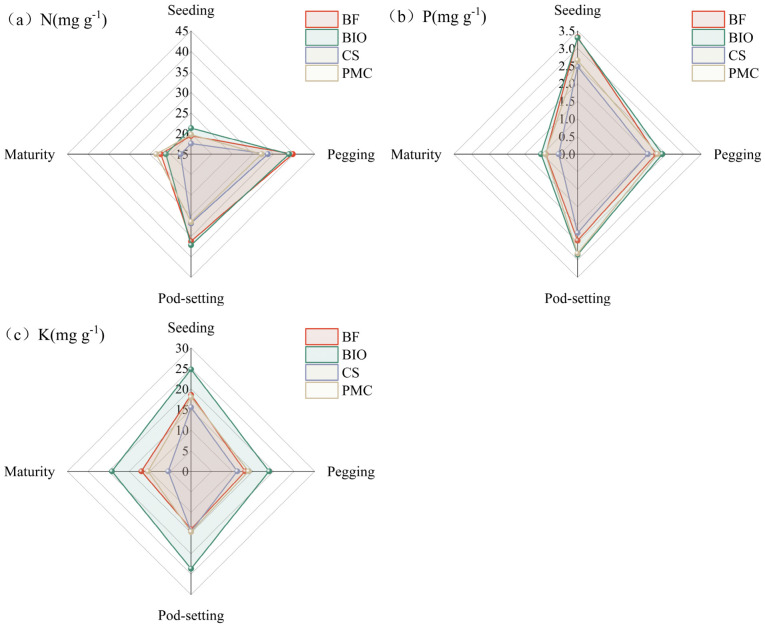
Leaf nutrient content in peanuts under four fertilization treatments at various growth stages. Data were collected from a long-term field micro-plot experiment established at Shenyang Agricultural University, China. Measurements were conducted at the seedling stage, flowering and pegging stage, pod-setting stage, and pod-filling to maturity stage. BF, biochar-based fertilizer; BIO, biochar + NPK fertilizer; CS, corn straw return + NPK fertilizer; PMC, pig manure compost + NPK fertilizer. (**a**–**c**) represent the nitrogen (*N*), phosphorus (*P*), and potassium (*K*) content in peanut leaves, respectively. Values are expressed as mg nutrient g^−1^ leaf dry matter.

As shown in Figure 5, BIO maintained relatively high leaf nitrogen (*N*), phosphorus (*P*), and potassium (*K*) contents across different growth stages. Leaf nitrogen content exhibited an increasing–decreasing trend, rising from the seedling stage to the pod-setting stage and then declining rapidly during the pod-filling to maturity stage. The lowest *N* content was observed under CS, reaching 17.5 mg g^−1^. Compared with CS and PMC, BF and BIO maintained higher leaf nitrogen contents across all growth stages, especially at the flowering and pegging and pod-setting stages, where they were significantly higher than those under the other treatments (*p* < 0.05). Differences in leaf phosphorus content among fertilization treatments were relatively small throughout most growth stages. From the seedling stage to the pod-setting stage, variation in leaf phosphorus content among treatments remained limited, whereas at the pod-filling to maturity stage, leaf phosphorus contents remained low across all treatments due to dilution by dry matter accumulation. The variation in leaf phosphorus content across peanut growth stages was unstable, with values ranging from as high as 24.8 mg g^−1^ to as low as 5.52 mg g^−1^. Among the treatments, BIO consistently maintained a relatively high leaf K content, particularly at the pod-filling to maturity stage, where it was 59–248% higher than those observed under the other treatments.

**Figure 6:**
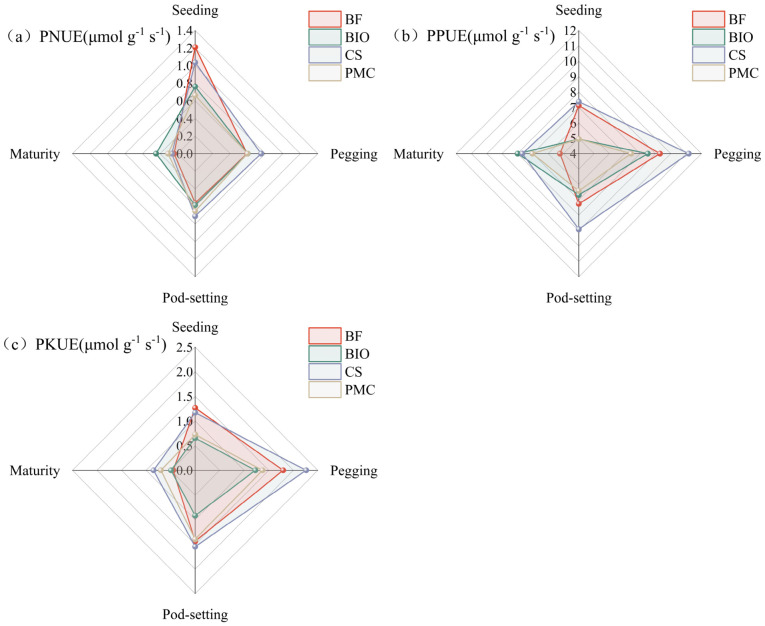
Photosynthetic nutrient use efficiency of peanut leaves under four fertilization treatments at various growth stages. Data were collected from a long-term field micro-plot experiment established at Shenyang Agricultural University, China. Measurements were conducted at the seedling stage, flowering and pegging stage, pod-setting stage, and pod-filling to maturity stage. BF, biochar-based fertilizer; BIO, biochar + NPK fertilizer; CS, corn straw return + NPK fertilizer; PMC, pig manure compost + NPK fertilizer. (**a**–**c**) represent the photosynthetic nitrogen (*N*), phosphorus (*P*), and potassium (*K*) use efficiency in peanut leaves, respectively. Units are μmol g^−1^ s^−1^ (micromoles per gram per second).

Photosynthetic nitrogen-, phosphorus-, and potassium-use efficiencies reflect the extent to which leaf *N*, *P*, and *K* promote photosynthesis. As shown in Figure 6, CS exhibited relatively high photosynthetic nutrient-use efficiencies across all growth stages, especially at the flowering and pegging stage, when photosynthetic nitrogen-, phosphorus-, and potassium-use efficiencies reached 0.75, 11.2, and 2.26, respectively, which were significantly higher than those under the other treatments (*p* < 0.05), with increases of 26–27%, 20–51%, and 26–84%, respectively. BF showed relatively high photosynthetic nutrient-use efficiency only at the seedling stage. In contrast, although BIO maintained high *N*, *P*, and *K* contents, it showed higher photosynthetic nitrogen- and phosphorus-use efficiencies only at the pod-filling to maturity stage compared with the other treatments, where they were significantly higher; overall, however, its photosynthetic nutrient-use efficiency remained relatively low. This indicates that peanut plants under BIO adopted a reserve-oriented strategy, in which nutrients absorbed during the early growth stage were preferentially allocated to the construction of the photosynthetic system and root development, and were then efficiently converted into photosynthetic output at the later stage. Although CS showed high nutrient-use efficiency during the early growth stage, its efficiency declined at the later stage due to nutrient depletion.

#### 3.3.2. Differences in Leaf N, P, and K Nutrient Ratios

Leaf nutrient ratios (*N*:*P*, *N*:*K* and *K*:*P*) were used to evaluate the nutrient status of peanut leaves during four growth stages. It should be noted that the threshold values adopted in this study were derived from previous studies on plant nutrient ratios and were used as comparative diagnostic references rather than absolute nutrient limitation criteria specifically established for peanut.

**Figure 7:**
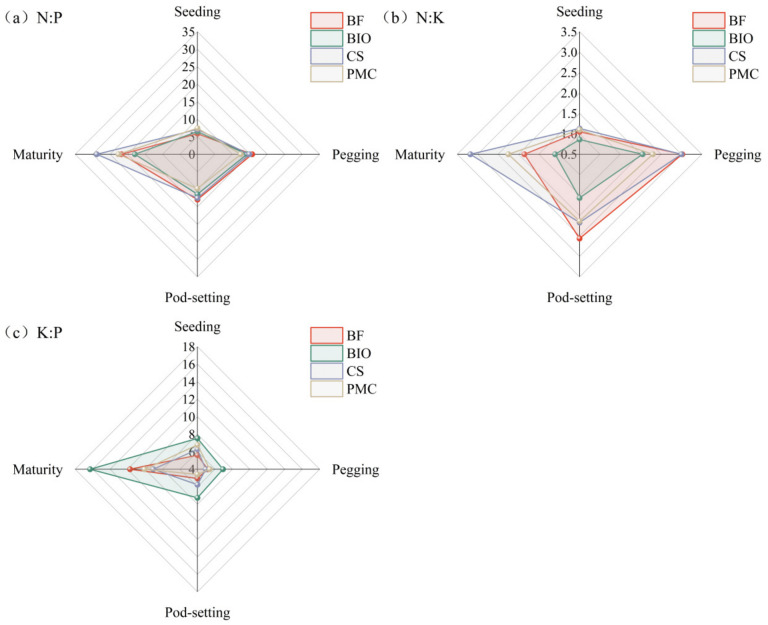
Leaf nutrient ratios under four fertilization treatments at various growth stages. Data were collected from a long-term field micro-plot experiment established at Shenyang Agricultural University, China. Measurements were conducted at the seedling stage, flowering and pegging stage, pod-setting stage, and pod-filling to maturity stage. BF, biochar-based fertilizer; BIO, biochar + NPK fertilizer; CS, corn straw return + NPK fertilizer; PMC, pig manure compost + NPK fertilizer. (**a**–**c**) represent the ratios of *N*:*P*, *N*:*K*, and *K*:*P* in peanut leaves, respectively.

As shown in Figure 7, based on the threshold values for the *N*:*P* nutrient ratio (*N*:*P* < 14 indicating nitrogen limitation and *N*:*P* > 16 indicating phosphorus limitation), peanut leaf *N*:*P* exhibited a similar pattern across growth stages under different fertilization treatments. The *N*:*P* ratios at the seedling and pod-setting stages were all lower than 14, suggesting a tendency towards nitrogen deficiency relative to phosphorus, whereas those at the flowering and pegging stage ranged between 14 and 16, suggesting a transitional stage of balanced nitrogen and phosphorus supply, with the exception of PMC, which showed a tendency towards phosphorus imbalance. At the pod-filling to maturity stage, the *N*:*P* ratios of all treatments exceeded 16, suggesting a shift towards phosphorus deficiency relative to nitrogen. Significant differences were observed among treatments (*p* < 0.05). BF showed a marked increase in *N*:*P* during the late growth stage, indicating a higher degree of phosphorus-related nutrient imbalance. PMC exhibited a strong nitrogen supply capacity at the pod-setting stage but showed a relatively more severe phosphorus-related nutrient imbalance at the later stage. In contrast, BIO showed a relatively balanced pattern overall. Specifically, the *N*:*P* ratio under BIO at the flowering and pegging stage was 13.5, suggesting a slight tendency towards nitrogen deficiency, which may be conducive to the regulation of reproductive growth. At the pod-filling to maturity stage, the *N*:*P* ratio under BIO (17.3) was significantly lower than that under CS (28.2), PMC (21.9), and BF (21.1), suggesting the lowest degree of phosphorus-related nutrient imbalance, a more coordinated nitrogen and phosphorus supply, and higher stability throughout the growth period. These results further suggest that nitrogen supply should be emphasized during the early and middle growth stages of peanut, whereas phosphorus regulation should be strengthened during the late growth stage.

Using the *N*:*K* nutrient ratio as a relative indicator of nutrient balance (*N*:*K* < 2.1 indicating nitrogen limitation and *N*:*K* > 2.1 indicating potassium limitation), the types of nutrient limitation in peanut differed significantly among treatments across growth stages (*p* < 0.05). At the seedling stage, the *N*:*K* ratios of all treatments were below 2.1, suggesting a relatively higher nitrogen demand compared with potassium. At the flowering and pegging stage, except for BIO, the *N*:*K* ratios of all other treatments exceeded 2.1, suggesting a tendency towards potassium-related nutrient imbalance. A similar pattern was observed at the pod-setting stage. At the pod-filling to maturity stage, the *N*:*K* ratio under BIO remained below 2.1, indicating a relatively balanced potassium nutritional status compared with the other treatments. Overall, except for BIO, all treatments showed an increasing tendency towards potassium-related nutrient imbalance after flowering, with CS showing the greatest potassium-related nutrient imbalance at the pod-filling to maturity stage, where the *N*:*K* ratio reached as high as 3.2. Therefore, BIO showed a clear advantage in maintaining a more balanced potassium nutritional status throughout the reproductive period.

Using the *K*:*P* nutrient ratio as a relative indicator of nutrient balance (*K*:*P* < 3.4 indicating potassium limitation), the *K*:*P* ratios of all treatments remained above 3.4 throughout all growth stages. The *K*:*P* ratio was relatively stable from the flowering stage to the pod-setting stage, but increased significantly after entering the pod-filling to maturity stage. In particular, BIO reached a *K*:*P* ratio as high as 16.0 during the late growth stage, indicating a strong tendency for potassium enrichment. In contrast, BF maintained relatively low *K*:*P* ratios across all growth stages, suggesting a relatively stronger phosphorus supply capacity or slower potassium accumulation. Overall, BIO maintained a more balanced potassium and phosphorus nutritional status during the late growth stage, thereby providing a favorable nutritional basis for sustained photosynthetic activity, thereby providing a nutritional basis for delaying senescence and maintaining photosynthesis.

### 3.4. Effects of Different Fertilization Treatments on Peanut Yield and Carbon Allocation

#### 3.4.1. Yield and Yield Components

As shown in Table 2, BIO produced the highest yield, reaching 7230.3 kg ha ^−1^, which was significantly higher than that of the other treatments. The yields of BF and PMC were 5964.1 and 6066.0 kg ha^−1^, respectively, whereas CS showed the lowest yield, at only 5622.4 kg ha^−1^. These results indicate that, compared with conventional organic materials, biochar addition significantly increased peanut yield. Although the yield under BF differed only slightly from that under PMC, it was still significantly higher than that under CS. The 100-pod weight, 100-kernel weight, and kernel percentage are also important components of yield. Based on these yield components, the higher yield under BIO was mainly associated with its significantly higher 100-pod weight, whereas its 100-kernel weight and kernel percentage were not consistently higher than those under all other treatments.

#### 3.4.2. Carbon Allocation Characteristics

**Figure 8:**
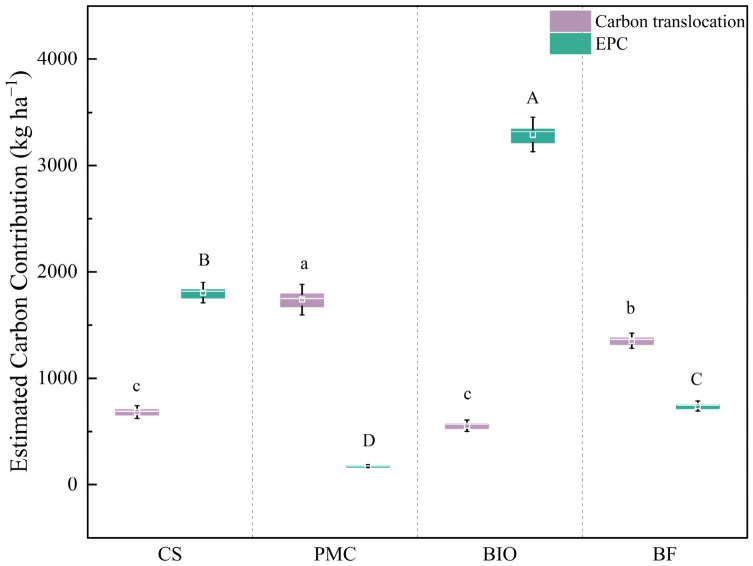
Estimated carbon translocation and estimated photosynthetic carbon contribution (EPC) to peanut pods under four fertilization treatments. Data were collected from a long-term field micro-plot experiment established at Shenyang Agricultural University, China. Measurements were conducted from the pod-setting stage to the pod-filling to maturity stage. BF, biochar-based fertilizer; BIO, biochar + NPK fertilizer; CS, corn straw return + NPK fertilizer; PMC, pig manure compost + NPK fertilizer. Different lowercase letters indicate significant differences in carbon translocation among fertilization treatments, whereas different uppercase letters indicate significant differences in EPC among fertilization treatments, as determined by one-way ANOVA followed by Duncan’s multiple range test (*p* < 0.05).

As shown in Figure 8, pod dry matter accumulation was assumed to be supported by two major sources: current photosynthetic assimilation occurring after pod setting and the remobilization of assimilates previously stored in vegetative organs before pod setting. Carbon translocation was estimated as the difference in stem and leaf dry weight between the pod-setting stage and the pod-filling to maturity stage, providing an indirect estimate of the apparent remobilization of stored assimilates from vegetative organs during this period. Estimated photosynthetic carbon contribution (EPC) was calculated as pod dry weight minus carbon translocation, representing an indirect proxy of the contribution of current photosynthates to pod biomass accumulation. Based on the estimated results shown in the figure, BIO exhibited a relatively higher estimated proportion of photosynthetic carbon, accounting for 85.6% of pod dry weight. In contrast, PMC and BF showed higher carbon translocation amounts, reaching 1737.6 and 1351.4 kg ha^−1^, respectively, which accounted for 64.7% and 90.9% of pod dry weight, suggesting a stronger dependence on remobilized assimilates from vegetative organs during pod development. These results suggest that the higher yield observed under BIO may be associated with sustained late-stage photosynthetic performance and a larger estimated contribution of current photosynthates to pod biomass accumulation, although direct carbon flux measurements were not performed in the present study.

#### 3.4.3. Effect Size Analysis of Biochar Treatment on Core Indicators of Peanut

Effect size analysis of key indicators at the pod-filling to maturity stage of peanut (Table 3) showed that, compared with the control treatments, BIO exhibited significant large effect sizes for most core indicators (Hedges’ g > 0.8), with 95% confidence intervals that did not include zero, confirming the overall stability and practical biological significance of the biochar effect. The results showed that BIO had the most pronounced effects on increasing leaf *K* content, optimizing the *N*:*P* ratio, and improving pod yield, with Hedges’ g values exceeding 0.8, indicating very large effects. In some comparisons, the Hedges’ g values for *Pn* were also higher than 0.8, and the corresponding 95% confidence intervals did not include zero, indicating that biochar significantly maintained the photosynthetic capacity of peanut leaves during the late growth stage. However, due to the limited sample size, the 95% confidence intervals for some comparisons included zero, including the comparison with PMC in the analysis of *Pn* and the comparisons with BF and CS in the analysis of SPAD. This suggests that the statistical stability of these differences was insufficient under the small-sample conditions of this study; nevertheless, the direction of the mean differences was clear, still indicating a potential positive effect of BIO on these indicators. Therefore, the finding that most core indicators in this study reached large effect sizes and had 95% confidence intervals that did not include zero confirms that biochar has a stable effect on improving photosynthetic performance, optimizing nutrient balance, and increasing yield.

### 3.5. Redundancy Analysis of Factors Associated with Peanut Yield Under Different Fertilization Treatments

**Figure 9:**
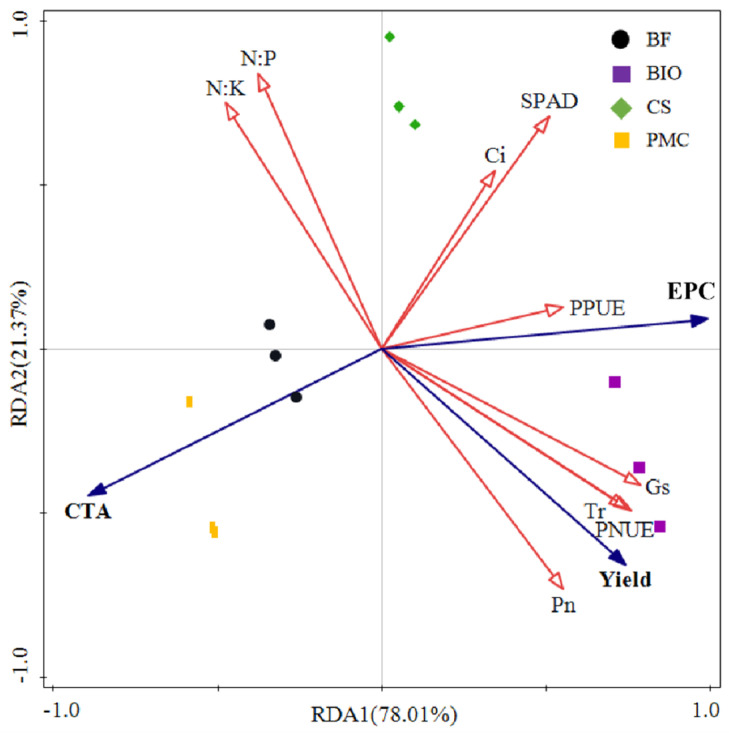
Redundancy analysis (RDA) showing associations among photosynthetic parameters, nutrient-related indicators, estimated photosynthetic carbon contribution (EPC), carbon translocation amount (CTA), and peanut yield. EPC was estimated from dry-matter differences rather than directly measured by isotope tracing or carbon flux techniques. BF, biochar-based fertilizer; BIO, biochar + NPK fertilizer; CS, corn straw return + NPK fertilizer; PMC, pig manure compost + NPK fertilizer. *Pn*, net photosynthetic rate; *Gs*, stomatal conductance; *Ci*, intercellular CO_2_ concentration; *Tr*, transpiration rate; SPAD, relative chlorophyll index; PNUE, photosynthetic nitrogen-use efficiency; PPUE, photosynthetic phosphorus-use efficiency; *N*:*P*, nitrogen-to-phosphorus ratio; *N*:*K*, nitrogen-to-potassium ratio.

According to the redundancy analysis (RDA) of yield, carbon translocation, photosynthetic carbon, and photosynthetic and nutrient-related factors at the pod-filling to maturity stage of peanut (Figure 9), together with the significance test results (Table 4), the first RDA axis explained approximately 78% of the variation, whereas the second axis explained approximately 21%. Together, the two axes accounted for about 99% of the total variation, indicating that the ordination adequately captured the relationships among variables. Yield showed positive associations with *Pn*, *Gs*, PNUE, PPUE, and SPAD, several of which were statistically significant according to permutation tests (*p* < 0.05), among which *Pn* and *Gs* were the core photosynthetic physiological factors driving yield improvement. The arrows of *Pn* and *Gs* were highly aligned with that of yield, suggesting that enhanced photosynthetic capacity and gas exchange capacity during the late growth stage were critical for peanut yield formation. In contrast, yield was clearly negatively correlated with *N*:*P* and *N*:*K*, indicating that imbalances in nitrogen–phosphorus and nitrogen–potassium nutrition inhibited pod yield accumulation. This further supports the conclusion that the high yield under BIO was partly attributable to its lower phosphorus and potassium limitations. In terms of the distribution patterns of different fertilization treatments, BIO was mainly clustered in the direction of yield and the positive core photosynthetic factors, including SPAD, *Pn*, and *Gs*, showing the strongest spatial association with yield-driving factors. This indicates that BIO significantly enhanced chlorophyll synthesis, photosynthetic rate, photosynthetic nutrient-use efficiency, and gas exchange parameters during the late growth stage of peanut, further confirming that BIO achieved a synergistic advantage characterized by high photosynthetic performance, low nutrient limitation, and high yield.

## 4. Discussion

### 4.1. Photosynthetic Physiological Basis for the Delay of Peanut Leaf Senescence by Long-Term Biochar Application

Biochar can alleviate drought stress by improving soil water availability and enhancing soil water-holding capacity, thereby maintaining stomatal opening and photosynthetic rate in crops [20]. Substantial evidence has shown that the porous structure of biochar increases soil available water content. Although this was not directly measured in the present study, differences in stomatal behavior may indirectly reflect a similar physiological response. Our results were highly consistent with this view: BIO maintained a relatively high net photosynthetic rate at the pod-filling to maturity stage, although no significant difference was observed compared with CS, and its stomatal limitation value showed a significant negative correlation with intercellular CO_2_ concentration, indicating a more optimized dynamic balance (Figure 1 and Figure 3). This suggests that stomatal factors may have contributed to the decline in photosynthetic rate in *Arachis hypogaea* (peanut) leaves during the pod-filling to maturity stage [21]. This may be because the addition of organic materials can improve soil structure and water retention capacity, thereby helping to alleviate abiotic stress [22]. These findings indicate that biochar, through its unique porous structure and surface properties, may play a key buffering role during the late growth stage of peanut, when soil water is likely to become a limiting factor due to intensified transpiration. By creating a more stable water environment for roots, biochar helps maintain the normal physiological function of leaf stomata.

Unlike BF, which exhibited typical stomatal limitation during the late growth stage, as characterized by high *Ls* and low *Ci*, BIO showed a more coordinated *Ls*–*Ci*–*Pn* relationship at the pod-filling to maturity stage. Biochar can alleviate water stress by regulating stomatal behavior and significantly improve net photosynthetic rate and water-use efficiency in soybean [23]; it can also maintain stomatal opening and reduce drought-induced damage to photosynthetic organs in rice [24]. A long-term field experiment similarly showed that continuous biochar application may enhance photosynthesis in soybean, reduce fertilizer input, and increase yield, with yield being positively correlated with photosynthetic indicators [25]. These findings suggest that, in addition to influencing stomatal behavior through water regulation, biochar may also maintain the photosynthetic capacity of mesophyll cells by delaying leaf senescence. This is consistent with recent findings showing that biochar application can affect gas exchange parameters and chlorophyll fluorescence in peanut leaves, increase photochemical quenching efficiency, and reduce non-photochemical quenching, thereby improving leaf photosynthetic function and delaying the decline in photosystem activity and the progression of leaf senescence [26]. Although senescence-related indicators were not directly measured in the present study, SPAD values (Figure 4) and *Pn* under BIO remained at relatively favorable levels at the pod-filling to maturity stage, which may indirectly suggest that biochar delayed the decline in photosystem function in peanut leaves. Meanwhile, this also suggests that biochar addition may improve plant nutrition and alleviate stress, enabling crop leaves to maintain higher SPAD value and thereby directly enhancing light-harvesting capacity [27]. Therefore, the promotion of late-stage photosynthetic performance in peanut by biochar may result from the synergistic effects of improved rhizosphere water conditions that optimize stomatal regulation and physiological and biochemical processes that delay leaf functional senescence, providing dual support for sustained carbon assimilation during the critical reproductive growth stage.

### 4.2. Biochar Optimizes Leaf Nutrient Balance by Alleviating Nutrient Limitation

As a soil amendment, one of the core functions of biochar is to regulate nutrient retention and cycling [28]. Biochar is characterized by a high nutrient adsorption capacity and the ability to delay nutrient release [29], which may have contributed to the observed plant nutrient status and photosynthetic performance, although soil water content, available nutrients, SOC, root traits, and nutrient fluxes were not measured in the present study. The present results are consistent with these previously proposed mechanisms. Throughout the entire growth period, leaf nitrogen, phosphorus, and potassium contents under BIO were significantly higher than those under the other treatments (Figure 5), suggesting that BIO was associated with improved nutrient status and a more sustained nutrient supply pattern, consistent with previous reports [30]. In contrast, CS consistently showed the lowest leaf nitrogen content and the lowest yield (Table 2), indicating that the readily available nutrients released through rapid straw decomposition were insufficient to sustain nutrient supply throughout the growth period. PMC showed relatively high nitrogen content at the early stage, but declined markedly at the later stage, accompanied by a substantial decrease in SPAD value, which may indicate that nutrient availability from pig manure compost was less synchronized with peanut nutrient demand during later growth stages. BF exhibited high nitrogen content and photosynthetic rate at the seedling stage, but lacked sufficient persistence at the later stage, suggesting that BF may provide relatively stronger support during early growth stages, but less sustained effects during later growth stages. This study also revealed the dynamics of nutrient use in peanut plants under biochar application. During the early and middle growth stages, higher nutrient contents were not synchronously converted into higher photosynthetic nutrient-use efficiencies (PNUE, PPUE, and PKUE), especially when compared with CS (Figure 6). This phenomenon of high nutrient status but relatively low use efficiency has rarely been systematically clarified in previous studies. These patterns may suggest a tendency towards temporary nutrient accumulation before later utilization under biochar application. During the vegetative growth stage, absorbed nutrients may have been preferentially allocated to the development of a more robust photosynthetic system and enhanced root growth, thereby reserving energy for subsequent reproductive growth rather than being immediately converted into photosynthetic output [31]. This interpretation was further associated with the significantly higher photosynthetic carbon under BIO at the pod-filling to maturity stage compared with the other treatments (Figure 8).

The advantage of this strategy became fully evident during the late reproductive growth stage. At the pod-filling to maturity stage, PNUE and PPUE under BIO showed a reversal pattern and were significantly higher than those under the other treatments (Figure 6), while the proportion of photosynthetic carbon in pod dry weight was also the highest (Figure 8). This may indicate that nutrients accumulated during earlier growth stages were more effectively utilized during the critical period of pod filling to support sustained photosynthesis. Although CS showed relatively high photosynthetic nutrient-use efficiency at the flowering and pegging stage, this advantage was not translated into sustained yield improvement at the later stage, further reflecting the limitation of rapidly available organic materials. This interpretation is further supported by the analysis of nutrient ratios. Ecological stoichiometry theory suggests that ratios such as *N*:*P* and *N*:*K* in plant tissues are key indicators of nutrient limitation and metabolic strategies [32]. Since Koerselman and Meuleman proposed the *N*:*P* threshold values (<14 indicating nitrogen limitation and >16 indicating phosphorus limitation) [33], this framework has been widely applied in wetland and terrestrial ecosystems. Güsewell further reviewed that *N*:*P* ratios vary among terrestrial plants and that these thresholds are not absolute [34]. Based on a meta-analysis of 197 fertilization experiments, Yan et al. found considerable uncertainty in the use of classical *N*:*P* thresholds for diagnosing plant nutrient limitation [35]. Despite these debates, previous studies have commonly used the following thresholds to assess plant nutrient limitation: *N*:*P* > 16 indicates phosphorus limitation, *N*:*P* < 14 indicates nitrogen limitation, and 14 < *N*:*P* < 16 indicates combined nitrogen and phosphorus limitation [36]. Similarly, *N*:*K* < 2.1 indicates predominant nitrogen limitation, whereas *N*:*K* > 2.1 indicates predominant potassium limitation; according to the threshold criterion for potassium limitation, *K*:*P* < 3.4 indicates that potassium is the main limiting element [37]. It should be noted that these nutrient ratio thresholds were primarily derived from studies on plant nutrient ratios and empirical observations across different plant species, rather than being specifically established for peanut. Therefore, in the present study, these thresholds were used as comparative indicators of nutrient balance and nutrient status among treatments and growth stages, rather than as absolute criteria for diagnosing nutrient limitation in peanut. Meanwhile, studies on biochar-related stoichiometry have also advanced. Previous research has shown that high-dose biochar addition may induce nitrogen dilution in plants [38]. It has been found that biochar application reduced soil carbon–nitrogen nutrient imbalance, whereas chemical fertilizer application alone aggravated this imbalance. From the perspective of soil microbial carbon–nitrogen stoichiometry, these findings provide scientific support for leaf nutrient stoichiometry studies and confirm the unique role of biochar in maintaining carbon–nitrogen balance [39]. In the present study, analysis based on the above thresholds showed that BIO maintained the most stable *N*:*P* ratio throughout the growth period and exhibited the lowest degree of phosphorus-related nutrient imbalance at the pod-filling to maturity stage (Figure 7). More importantly, its *N*:*K* ratio remained consistently below 2.1 throughout the growth period, whereas the other treatments generally showed a stronger tendency towards potassium-related nutrient imbalance after flowering. This result may have practical implications for nutrient management in peanut production. Peanut is a potassium-loving crop that is highly sensitive to phosphorus and potassium supply during the middle and late growth stages, and conventional fertilization often fails to maintain nutrient supply at later stages [40]. Previous studies have suggested that biochar may enhance potassium retention and improve phosphorus availability through its physicochemical properties [41]. Consistent with these observations, the BIO treatment in the present study maintained a more balanced nutrient status during the reproductive stage, which may have contributed to reduced phosphorus- and potassium-related nutrient imbalances. However, because soil water content, soil organic carbon, available nutrient pools, root traits, and nutrient fluxes were not quantified, these soil-related mechanisms should be regarded as plausible explanations rather than demonstrated processes.

It should be noted that the four organic amendments used in this study differed not only in biochar content but also in carbon input, C ratio, decomposition characteristics, and nutrient-release dynamics. In addition, soil water content, soil organic carbon, available nutrient pools, root traits, and nutrient fluxes were not quantified in the present study. Therefore, potential soil-mediated mechanisms discussed above should be regarded as plausible explanations rather than direct evidence of the processes responsible for the observed treatment responses. Therefore, the observed differences among treatments cannot be attributed solely to the biochar component itself. Instead, they likely reflect the combined effects of amendment quality, nutrient supply patterns, and carbon stabilization processes under long-term field conditions. Although the BIO treatment generally showed better physiological performance and higher yield formation, the present field experiment was not designed to isolate the individual contribution of biochar from other amendment-related factors. Future studies using more controlled amendment compositions or isotope-tracing approaches would help clarify the specific mechanisms underlying the observed treatment responses.

### 4.3. Sustained Photosynthesis and Alleviated Nutrient Limitation Synergistically Drive Biochar-Induced Yield Improvement

The results of this study indicated that the BIO treatment maintained higher photosynthetic performance during the pod-filling to maturity stage and exhibited a more balanced nutrient status in leaves. The combined occurrence of these physiological and nutritional characteristics was associated with the higher yield observed under BIO treatment. Relationships revealed by carbon allocation estimates and redundancy analysis further supported this association, although the underlying mechanisms were not directly examined in the present study.

In this study, BIO not only maintained higher net photosynthetic rate (*Pn*) and SPAD values at the pod-filling to maturity stage, but also maintained a relatively low *N*:*K* ratio throughout the reproductive period. Meanwhile, its *N*:*P* ratio remained relatively stable, and a more balanced nutrient status with respect to phosphorus nutrition was observed during the late growth stage, suggesting a coordinated pattern involving relatively high photosynthetic capacity and improved nutrient balance. Carbon allocation estimates further suggested that this coordinated pattern may have been associated with more efficient biomass accumulation. Under BIO, photosynthetic carbon, estimated as the apparent contribution of newly assimilated photosynthates to pod dry matter from the pod-setting stage to the pod-filling to maturity stage, accounted for the highest proportion of pod dry weight, whereas carbon translocation, representing the redistribution of carbon reserves stored in vegetative organs, was relatively low (Figure 8). Previous studies have shown that biochar can promote the allocation of photosynthates to grains [42], and Yang et al. found that sustained photosynthesis during the late growth stage under biochar application provides a more direct and efficient carbon source for grain filling [43]. The performance of BIO in the present study was generally consistent with these findings: maintenance of photosynthetic capacity at the late growth stage was associated with a higher estimated contribution of current photosynthates to pod biomass accumulation and a reduced apparent reliance on remobilized assimilates from vegetative organs.

Redundancy analysis further showed that yield was significantly positively correlated with *Pn*, *Gs*, SPAD, PNUE, and PPUE, but negatively correlated with *N*:*P* and *N*:*K* ratios (Figure 9, Table 4). Previous studies have shown that maximum net photosynthetic rate is positively correlated with leaf *P* content but negatively correlated with the *N*:*P* ratio, indicating that nutrient imbalance may weaken the potential enhancement of photosynthetic capacity [44,45]. These findings suggest that higher photosynthetic performance, improved nutrient balance, and a higher estimated photosynthetic carbon contribution were closely associated with yield formation during the late growth stage. Liu et al., using quantitative ^13^C isotope tracing, found that biochar application increased photosynthetic carbon assimilation by improving nitrogen-use efficiency, thereby enhancing crop productivity [46]. In the present study, BIO showed the highest proportion of photosynthetic carbon in pod dry weight at the pod-filling to maturity stage. In contrast, CS exhibited the highest *N*:*K* ratio during the late growth stage, suggesting a higher degree of potassium-related nutrient imbalance, with an *N*:*K* ratio as high as 3.2, and an excessively high proportion of carbon translocation, which may indicate relatively lower late-stage photosynthetic contribution and stronger apparent reliance on carbon export from stems and leaves. PMC showed intensified phosphorus limitation, whereas BF showed relatively stronger early-stage responses but less sustained effects during later growth stages; neither treatment achieved sustained coupling among photosynthesis, nutrient balance, and carbon allocation. It should also be noted that, in the effect size analysis (Table 3), the 95% confidence intervals for some comparisons, including *Pn* in BIO vs. PMC and SPAD in BIO vs. BF/CS, crossed zero due to the limited sample size (*n* = 3). However, the direction of the mean differences was clear, and most Hedges’ g values were higher than 0.8. Thompson noted that, under small-sample conditions, confidence intervals are more likely to cross zero, but Hedges’ g values higher than 0.8 still indicate large effect sizes [47]. Other scholars have also emphasized that biological significance should not be judged solely by whether the confidence interval includes zero, but should be evaluated comprehensively by considering effect size magnitude and the direction of mean differences [48]. Therefore, these results should be interpreted cautiously and may indicate potentially positive effects of biochar, although additional studies with larger sample sizes are required to improve statistical power and further verify these patterns. It should be noted that the RDA was performed using a relatively limited number of observations (*n* = 12) in relation to the number of explanatory variables included in the model. Although forward selection and permutation testing were used to reduce model overfitting and evaluate statistical significance, correlations among explanatory variables may still influence ordination results. Therefore, the RDA should be interpreted primarily as an exploratory tool for identifying potential associations among photosynthetic traits, nutrient status, carbon allocation characteristics, and yield formation, rather than as definitive evidence of causal relationships. Future studies with larger sample sizes and independent validation datasets are needed to further confirm these relationships. In summary, long-term biochar application was associated with the maintenance of higher photosynthetic performance and improved nutrient balance during the late growth stage, thereby promoting a larger apparent photosynthetic carbon contribution to pods and ultimately contributing to sustained yield improvement under long-term field conditions.

## 5. Conclusions

Long-term biochar application (BIO treatment) was associated with relatively higher leaf physiological performance during the late growth stage of *Arachis hypogaea* (peanut), as reflected by higher net photosynthetic rate, SPAD value, and a more coordinated relationship between stomatal limitation and intercellular CO_2_ concentration at the pod-filling to maturity stage. BIO was also associated with a relatively more balanced nutrient status during the late growth stage, particularly with respect to potassium and phosphorus nutrition. Under long-term field conditions, BIO achieved the highest yield (7230.3 kg ha^−1^) among all treatments. The higher yield observed under BIO may be related to the combined occurrence of sustained late-stage photosynthetic performance and improved nutrient coordination. These findings provide new insights into how long-term biochar application may contribute to improved peanut productivity under long-term field conditions.

## Figures and Tables

**Table 1:** Chemical characteristics of the organic amendments used in the long-term field micro-plot experiment conducted at the National Peanut Industry Technology System Soil and Fertilizer Long-term Experimental Station of Shenyang Agricultural University (Shenyang, Liaoning Province, China).

Treatment	C (%)	N (%)	P (%)	K (%)	C/N
BF	7.73	11.0	5.67	10.8	0.70
BIO	33.3	0.50	0.37	0.49	66.6
CS	44.4	1.01	0.09	0.77	44.4
PMC	29.2	1.44	0.47	0.78	24.3

Notes: BF, biochar-based fertilizer; BIO, biochar + NPK fertilizer; CS, maize straw return + NPK fertilizer; PMC, pig manure compost + NPK fertilizer. This experiment was conducted in a long-term field micro-plot system established in 2009 at Shenyang Agricultural University. Units are given on a dry weight basis.

**Table 2:** Pod yield and yield components of *Arachis hypogaea* (peanut) under four fertilization treatments in the long-term field micro-plot experiment conducted at Shenyang Agricultural University in 2024.

Treatments	Pod Yield (kg ha^−1^)	100-Pod Weight (g)	100-Kernel Weight (g)	Kernel Percentage (%)
BF	5964.1 ± 91.7 bc	172.5 ± 0.34 b	75.5 ± 0.25 a	69.0 ± 0.24 b
BIO	7230.3 ± 165.2 a	177.4 ± 0.32 a	74.5 ± 0.27 b	69.5 ± 0.26 b
CS	5622.4 ± 80.3 c	161.3 ± 0.44 c	74.2 ± 0.22 b	69.7 ± 0.20 b
PMC	6066.0 ± 149.0 b	149.6 ± 0.42 d	70.7 ± 0.24 c	72.2 ± 0.14 a

Notes: BF, biochar-based fertilizer; BIO, biochar + NPK fertilizer; CS, corn straw return + NPK fertilizer; PMC, pig manure compost + NPK fertilizer. Data are presented as mean ± standard error (*n* = 3). Different lowercase letters indicate significant differences in the same indicator among treatments, as determined by one-way analysis of variance (ANOVA) followed by Duncan’s multiple range test (*p* < 0.05).

**Table 3:** Effect size analysis of core indicators between biochar treatment (BIO) and control treatments.

Parameter	Comparison	MD (95% CI)	*p*	Hedges’ g
Pn	BIO vs. BF	4.03 (2.04, 6.03)	0.005	3.68
	BIO vs. CS	4.73 (2.14, 7.32)	0.016	5.06
	BIO vs. PMC	2.13 (−0.43, 4.69)	0.071	2.27
SPAD	BIO vs. BF	1.12 (−1.05, 3.29)	0.225	0.94
	BIO vs. CS	−1.81 (−5.11, 1.50)	0.202	−1.00
	BIO vs. PMC	2.71 (0.28, 5.14)	0.036	2.02
K	BIO vs. BF	7.14 (5.83, 8.45)	<0.001	9.87
	BIO vs. CS	13.67 (12.49, 14.86)	<0.001	21.00
	BIO vs. PMC	8.75 (7.40, 10.10)	<0.001	11.74
N:P	BIO vs. BF	−3.81 (−6.21, −1.41)	0.012	−2.88
	BIO vs. CS	−10.89 (−13.69, −8.10)	<0.001	−7.06
	BIO vs. PMC	−4.67 (−7.10, −2.23)	0.006	−3.48
Yield	BIO vs. BF	1266.19 (741.62, 1790.76)	0.003	4.38
	BIO vs. CS	1607.89 (1097.95, 2117.83)	0.001	5.72
	BIO vs. PMC	1164.31 (546.71, 1781.92)	0.006	3.42

Notes: BIO, biochar + NPK fertilizer; BF, biochar-based fertilizer; CS, corn straw return + NPK fertilizer; PMC, pig manure compost + NPK fertilizer. *Pn*, net photosynthetic rate; SPAD, relative chlorophyll index; *N*:*P*, nitrogen-to-phosphorus ratio. MD represents the mean difference between treatment means; Hedges’ g is a small-sample corrected effect size; CI represents the 95% confidence intervals. Comparisons whose confidence interval crosses zero indicate limited statistical stability under small sample conditions and are not statistically significant. Criteria for effect size interpretation: g ≥ 0.8 indicates a large effect, 0.5 ≤ g < 0.8 indicates a medium effect, and 0.2 ≤ g < 0.5 indicates a small effect.

**Table 4:** Significance test results.

Index	Explains %	Contribution %	F	*p*
Gs	52.4	52.7	11.0	0.006 **
SPAD	27.9	28	12.7	0.002 **
N:P	6.0	6.0	3.5	0.06
Pn	5.7	5.7	5.0	0.024 *
PNUE	5.3	5.3	11.7	0.004 **
PPUE	1.6	1.6	7.4	0.032 *
Ci	0.3	0.3	1.2	0.336
Tr	0.2	0.2	1.2	0.338
N:K	0.1	0.1	0.5	0.546

Notes: *Gs*, stomatal conductance; SPAD, relative chlorophyll index; *N*:*P*, nitrogen-to-phosphorus ratio; *Pn*, net photosynthetic rate; PNUE, photosynthetic nitrogen-use efficiency; PPUE, photosynthetic phosphorus-use efficiency; *Ci*, intercellular CO_2_ concentration; *Tr*, transpiration rate; *N*:*K*, nitrogen-to-potassium ratio. F, F statistic in permutation significance test. ** indicates extremely significant correlation (*p* < 0.01); * indicates significant correlation (*p* < 0.05).

## Data Availability

The data that support the findings of this study are available from the authors upon reasonable request. The data are not publicly available due to privacy and ethical restrictions.

## References

[1] Zhang M., Wang L.F., Wan Y.S., Liu F.Z., Zhang K. (2017). Rational Nitrogen Strategies Can Improve Peanut Source Supply Capacity and Pod Yield. Agron. J..

[2] Liu Z., Gao F., Yang J., Zhen X., Li Y., Zhao J., Li J., Qian B., Yang D., Li X. (2019). Photosynthetic Characteristics and Uptake and Translocation of Nitrogen in Peanut in a Wheat–Peanut Rotation System Under Different Fertilizer Management Regimes. Front. Plant Sci..

[3] Kumar A., Bhattacharya T., Shaikh W.A., Roy A., Chakraborty S., Vithanage M., Biswas J.K. (2023). Multifaceted applications of biochar in environmental management: A bibliometric profile. Biochar.

[4] Mahmoud A.W.M., Samy M.M., Sany H., Eid R.R., Rashad H.M., Abdeldaym E.A. (2022). Nanopotassium, Nanosilicon, and Biochar Applications Improve Potato Salt Tolerance by Modulating Photosynthesis, Water Status, and Biochemical Constituents. Sustainability.

[5] Bloukounon-Goubalan A.Y., Saïdou A., Obognon N., Amadji G.L., Igué A.M., Clottey V.A., Kenis M. (2019). Decomposition and nutrient release pattern of animal manures biodegraded by fly larvae in Acrisols. Can. J. Soil Sci..

[6] Guo M., Song W., Tian J. (2020). Biochar-Facilitated Soil Remediation: Mechanisms and Efficacy Variations. Front. Environ. Sci..

[7] Yin H.J., Zhao W.Q., Li T., Cheng X.Y., Liu Q. (2018). Balancing straw returning and chemical fertilizers in China: Role of straw nutrient resources. Renew. Sustain. Energy Rev..

[8] Doyeni M.O., Stulpinaite U., Baksinskaite A., Suproniene S., Tilvikiene V. (2021). The Effectiveness of Digestate Use for Fertilization in an Agricultural Cropping System. Plants.

[9] Wu Z.Z., Liu J.X., Nie J.M., Liang C., Guo S.M., Zhou C.C., Huang Y.C., Wang S. (2025). Co-Incorporation of Controlled-Release Urea and Conventional Urea Enhances Rice Yield, Economic Benefits, and Nitrogen Use Efficiency in Saline–Alkali Paddy Fields. Agronomy.

[10] Li Y.Y., Wang J.X., Wang L.Q. (2024). Seasonal variations in C/N/P/K stoichiometric characteristics in different plant organs in the various forest types of Sygera Mountain. Front. Plant Sci..

[11] Li R.R., Lu Y., Wan F.X., Wang Y.M., Pan X.C. (2018). Impacts of a High Nitrogen Load on Foliar Nutrient Status, N Metabolism, and Photosynthetic Capacity in a *Cupressus lusitanica* Mill. Plantation. Forests.

[12] Xu X.X., Zhang X., Ni W., Liu C.L., Qin H.H., Guan Y.F., Liu J.Q., Feng Z.Q., Xing Y., Tian G. (2024). Nitrogen–potassium balance improves leaf photosynthetic capacity by regulating leaf nitrogen allocation in apple. Hortic. Res..

[13] Hou J., Pugazhendhi A., Sindhu R., Vinayak V., Thanh N.C., Brindhadevi K., Chi N.T.L., Yuan D. (2022). An assessment of biochar as a potential amendment to enhance plant nutrient uptake. Environ. Res..

[14] Neththasinghe N., Dissanayaka D., Karunarathna A. (2023). Rhizosphere nutrient availability and nutrient uptake of soybean in response to biochar application. J. Plant Nutr..

[15] Rohitha D.S., Mamatha B., Reddy K.M.S., Prakasha H.C., Desai N. (2020). Influence of Different Levels of Coconut Shell Biochar on Nutrients Concentration and Nutrient Uptake by Soybean. Int. J. Curr. Microbiol. Appl. Sci..

[16] Chen R.R., Wang X.T. (2022). BOOK REVIEW: Analytical methods for soil and agro-chemistry (in Chinese). Edited by H. Z. Zhu, P. A. He, C. Z. Chen, H. M. Zhou, D. C. Su, J. M. Xu, H. Y. Qin, S. D. Bao, R. K. Lu S. H. Jiang Soil Science Society of China Beijing, China Agricultural Science and Technology Press, 2000, pp. 638. ISBN: 9787801199256. Eur. J. Soil Sci..

[17] Varley J. (1966). Automatic methods for the determination of nitrogen, phosphorus and potassium in plant material. Analyst.

[18] Nakagawa S., Cuthill I.C. (2007). Effect size, confidence interval and statistical significance: A practical guide for biologists. Biol. Rev..

[19] Ter Braak C.J., Verdonschot P.F. (1995). Canonical correspondence analysis and related multivariate methods in aquatic ecology. Aquat. Sci..

[20] Tanure M.M.C., da Costa L.M., Huiz H.A., Fernandes R.B.A., Cecon P.R., Junior J.D.P., da Luz J.M.R. (2019). Soil water retention, physiological characteristics, and growth of maize plants in response to biochar application to soil. Soil Tillage Res..

[21] Gao Y., Tang Z.H., Xia H.Q., Sheng M.F., Liu M., Pan S.Y., Li Z.Y., Liu J.R. (2021). Potassium Fertilization Stimulates Sucrose-to-Starch Conversion and Root Formation in Sweet Potato (*Ipomoea batatas* (L.) Lam.). Int. J. Mol. Sci..

[22] Liu Y.R., Lan X.J., Hou H.Q., Ji J.H., Liu X.M., Lv Z.Z. (2024). Multifaceted Ability of Organic Fertilizers to Improve Crop Productivity and Abiotic Stress Tolerance: Review and Perspectives. Agronomy.

[23] Wang H.H., Ren T.B., Müller K., Van Zwieten L., Wang H.L., Feng H.L., Xu C.S., Yun F., Ji X.M., Yin Q.Y. (2021). Soil type regulates carbon and nitrogen stoichiometry and mineralization following biochar or nitrogen addition. Sci. Total Environ..

[24] Zoz T., Fiorese D.A., Pivetta L.A., Zoz A., Zoz J., Zuffo A.M. (2018). Phosphorus and potassium fertilization in creeping peanut. Sci. Agrar..

[25] Wang P.J., Liu Q., Fan S.L., Wang J., Mu S.G., Zhu C.B. (2023). Combined Application of Desulfurization Gypsum and Biochar for Improving Saline-Alkali Soils: A Strategy to Improve Newly Reclaimed Cropland in Coastal Mudflats. Land.

[26] Wang S.J., Zheng J.L., Wang Y.J., Yang Q.F., Chen T.T., Chen Y.L., Chi D.C., Xia G.M., Siddique K.H.M., Wang T.L. (2021). Photosynthesis, Chlorophyll Fluorescence, and Yield of Peanut in Response to Biochar Application. Front. Plant Sci..

[27] Tu Q.F., Tang S.Y., Huang S.C. (2025). Mitigation of salinity stress via improving growth, chlorophyll contents and antioxidants defense in sunflower with Bacillus pumilis and biochar. Sci. Rep..

[28] Tang K.H.D. (2025). Biochar Amendments for Soil Restoration: Impacts on Nutrient Dynamics and Microbial Activity. Environments.

[29] Lee Y.-E., Jeong Y., Shin D.-C., Ahn K.-H., Jung J.-H., Kim I.-T. (2021). Fabrication of Mg-Doped Sargassum Biochar for Phosphate and Ammonium Recovery. Sustainability.

[30] Li T., Mei C.G., Zhao C.C., Wu J., Jia S.Y., Liu W.W., Chen J., Li X.Y., Cheng B.J., Zhao Z.N. (2026). Granular Mg-enriched biochar-based compound fertilizer: Enhanced slow-release performance and effects on maize growth under reduced application rates. Ind. Crops Prod..

[31] Wang X.Z., Wu M., Sun C.B., Liu M., Yang L.Y., Liang H.Y., Wu Q., Shen P. (2024). Biochar distribution mode in soil affects the vegetative peanut growth, nitrogen uptake and nitrogen-fixing bacteria activity. Plant Soil Environ..

[32] Elser J.J. (2003). Biological stoichiometry: A theoretical framework connecting ecosystem ecology, evolution, and biochemistry for application in astrobiology. Int. J. Astrobiol..

[33] Koerselman W., Meuleman A.F. (1996). The vegetation N: P ratio: A new tool to detect the nature of nutrient limitation. J. Appl. Ecol..

[34] Güsewell S. (2004). N:P ratios in terrestrial plants: Variation and functional significance. New Phytol..

[35] Yan Z.B., Tian D., Han W.X., Tang Z.Y., Fang J.Y. (2017). An assessment on the uncertainty of the nitrogen to phosphorus ratio as a threshold for nutrient limitation in plants. Ann. Bot..

[36] Wright I.J., Reich P.B., Westoby M., Ackerly D.D., Baruch Z., Bongers F., Cavender-Bares J., Chapin T., Cornelissen J.H., Diemer M. (2004). The worldwide leaf economics spectrum. Nature.

[37] Olde Venterink H., Wassen M.J., Verkroost A., De Ruiter P. (2003). Species richness–productivity patterns differ between N-, P-, and K-limited wetlands. Ecology.

[38] Gale N.V., Thomas S.C. (2019). Dose-dependence of growth and ecophysiological responses of plants to biochar. Sci. Total Environ..

[39] Gonçalves M.A.F., da Silva B.R.S., Nobre J.R.C., Batista B.L., da Silva Lobato A.K. (2024). Biochar mitigates the Harmful effects of Drought in soybean through changes in Leaf Development, Stomatal Regulation, and Gas Exchange. J. Soil Sci. Plant Nutr..

[40] Zhang K.K., Han X.M., Fu Y.F., Khan Z., Zhang B.J., Bi J.G., Hu L.Y., Luo L.J. (2024). Biochar coating promoted rice growth under drought stress through modulating photosynthetic apparatus, chloroplast ultrastructure, stomatal traits and ROS homeostasis. Plant Physiol. Biochem..

[41] Wu D., Zhang Y.X., Gu W.Q., Feng Z.B., Xiu L.Q., Zhang W.M., Chen W.F. (2024). Long term co-application of biochar and fertilizer could increase soybean yield under continuous cropping: Insights from photosynthetic physiology. J. Sci. Food Agric..

[42] Qian Z., Zhang H.-J., Xue A. (2019). Effect of biochar on grain yield and leaf photosynthetic physiology of soybean cultivars with different phosphorus efficiencies. J. Integr. Agric..

[43] Yang Y.F., Ahmed W., Wang G., Ye C.H., Li S.C., Zhao M.W., Zhang J.H., Wang J.J., Salmen S.H., Wu L.Z. (2024). Transcriptome profiling reveals the impact of various levels of biochar application on the growth of flue-cured tobacco plants. BMC Plant Biol..

[44] Burns S.L., Pérez C.A., Arturi M.F., Goya J.F., Graciano C. (2011). Relationship Between Height Growth and Foliar Nutrient N and P Concentration in a Eucalyptus grandis Plantation in Northeastern Argentina. J. Sustain. For..

[45] Tian R.P., Li L.Y., Zhang D.J., Zhang J., Wang C.K., Quan X.K. (2024). Response of Photosynthetic Capacity to Climate Warming and Its Variation among 11 Provenances of Dahurian Larch (*Larix gmelinii*). Forests.

[46] Liu Z.W., Wu X.L., Li S.X., Liu W., Bian R.J., Zhang X.H., Zheng J.F., Drosos M., Li L.Q., Pan G.X. (2021). Quantitative assessment of the effects of biochar amendment on photosynthetic carbon assimilation and dynamics in a rice–soil system. New Phytol..

[47] Thompson B. (2007). Effect sizes, confidence intervals, and confidence intervals for effect sizes. Psychol. Sch..

[48] Thompson B. (2008). Computing and interpreting effect sizes, confidence intervals, and confidence intervals for effect sizes. Best Practices in Quantitative Methods.

